# Supplementary material for: A scoping review of the electronic collection and capture of patient reported outcome measures for children and young people in the hospital setting
Source: PLOS Digit Health. 2025 Jan 6;4(1):e0000704. doi: 10.1371/journal.pdig.0000704 (PMC11703060; doi:10.1371/journal.pdig.0000704)
Supplement: S1 File — (DOCX) [file pdig.0000704.s001.docx]

# Supplementary File 1: Full inclusion and exclusion criteria

| Inclusion Criteria | Exclusion Criteria |
| --- | --- |
| Population  CYP from birth to 25 years old, or their proxies, treated in paediatric settings (including neonatal and adolescent).  If transitioning from children to adult services, patients are included if they are still in the paediatric setting. | Population  CYP treated in adult hospital settings (inpatient or outpatient)  Adults (aged over 25) |
| Concept  PROM use including:   - QOL/HRQoL measures - Disease-specific measures | Concept   - Research trials where PROMs data are outcomes measured – e.g., trials/studies where PROMs are measured in non-routine care - Developing or validating PROM - Only includes PREM use |
| Context  Routine clinical care for CYP in paediatric hospital care (including neonatal) inpatient or outpatient service within an adult hospital or children’s hospital, including:   - Medical and surgical - Emergency care (including in an emergency department providing paediatric services within an adult hospital) - Transition care (in paediatric setting) - Intensive care - Neonatal care - Paediatric outreach - Virtual paediatric hospital clinics - Specialist adolescent and young adult services (inpatient and outpatient) where data from patients under the age of 25 years can be separated from those over the age of 25 years. | Context   - Primary care - Adult emergency department setting with no paediatric service or professional input. - Adult inpatient or outpatient settings - Schools - Homes (unless part of paediatric outreach or virtual paediatric hospital clinics) - Hospices - Maternity services (including those caring AYAs) - Community clinics - Dental care |
