# Supplementary material for: A scoping review of the electronic collection and capture of patient reported outcome measures for children and young people in the hospital setting
Source: PLOS Digit Health. 2025 Jan 6;4(1):e0000704. doi: 10.1371/journal.pdig.0000704 (PMC11703060; doi:10.1371/journal.pdig.0000704)
Supplement: S2 File — (DOCX) [file pdig.0000704.s002.docx]

# Supplementary File 2: Example of search strategy (Medline)

| # | Searches | Results |
| --- | --- | --- |
| 1 | patient reported outcome measures/ | 7520 |
| 2 | PREM*.mp. | 377661 |
| 3 | (experience adj measure).mp. | 109 |
| 4 | ((friends and family test) or FFT).mp. | 2822 |
| 5 | (PROMs or PROM or PROMIS).mp. | 7006 |
| 6 | (patient reported adj (outcome* or experience* or treatment outcome* or indicator*)).mp. | 25341 |
| 7 | (patient adj (outcome assessment* or outcome measure* or survey* or questionnaire*)).mp. | 10789 |
| 8 | patient cent* outcome measure.mp. | 52 |
| 9 | patient experience questionnaire.mp. | 78 |
| 10 | (Self-report* adj (measure* or outcome*)).mp. | 15488 |
| 11 | (parent-report* adj (measure* or outcome* or experience*)).mp. | 659 |
| 12 | (quality of life or QoL or health-related quality of life or HRQoL or HRQL or health status).mp. | 495900 |
| 13 | 2 or 3 or 4 or 5 or 6 or 7 or 8 or 9 or 10 or 11 or 12 | 904901 |
| 14 | 1 or 13 | 904901 |
| 15 | Pediatrics/ | 54962 |
| 16 | adolescent/ or exp child/ or exp infant/ | 3628448 |
| 17 | (child* or adolescen* or teen* or youth* or young person* or young people* or infan* or toddler* or baby or babies).mp. | 4189308 |
| 18 | p?ediatric*.mp. | 427591 |
| 19 | 15 or 16 or 17 or 18 | 4254091 |
| 20 | 14 and 19 | 267046 |
| 21 | (clinical care or clinical practice or clinical implementation or clinical service* or clinical team*).mp. | 230335 |
| 22 | exp Patients/ | 68138 |
| 23 | (hospital patient* or inpatient* or outpatient*).mp. | 299696 |
| 24 | 22 or 23 | 328372 |
| 25 | 21 or 24 | 549106 |
| 26 | 20 and 25 | 11441 |
| 27 | 26 not clinical trial.pt. | 10951 |
| 28 | limit 27 to (english language and yr="2008 -Current") | 7559 |
| 29 | exp Animals/ | 23826038 |
| 30 | Humans/ | 19036693 |
| 31 | 29 not 30 | 4789345 |
| 32 | 28 not 31 | 7551 |
