# Supplementary material for: A scoping review of the electronic collection and capture of patient reported outcome measures for children and young people in the hospital setting
Source: PLOS Digit Health. 2025 Jan 6;4(1):e0000704. doi: 10.1371/journal.pdig.0000704 (PMC11703060; doi:10.1371/journal.pdig.0000704)
Supplement: S3 File — (DOCX) [file pdig.0000704.s003.docx]

# Supplementary File 3: Extraction form

| Types of evidence source |  |
| --- | --- |
| Evidence source Details and Characteristics | |
| Citation details |  |
| Author/s |  |
| Year of publication |  |
| Country |  |
| Method of PROMs and PREMs collection |  |
| Participants (details e.g., age/sex and number) |  |
| Details/Results extracted from source of evidence | |
| PROM/s reported (including tool details) |  |
| Context/setting (e.g. ward, emergency care) |  |
| Key findings in relation to:   1. How PROMs are used. 2. How are PROMs data applied in clinical practice. 3. Barriers and facilitators. 4. Data utilised towards the development of health care services. 5. Patient groups for whom PROMs are/are not an integral part of routine care provision. 6. Reports from CYP themselves vs proxies (parent/carer). 7. Data capture and access of ePROMs/ePROMs results for different demographic breakdowns. |  |
