# Supplementary material for: A scoping review of the electronic collection and capture of patient reported outcome measures for children and young people in the hospital setting
Source: PLOS Digit Health. 2025 Jan 6;4(1):e0000704. doi: 10.1371/journal.pdig.0000704 (PMC11703060; doi:10.1371/journal.pdig.0000704)
Supplement: S4 File — (DOCX) [file pdig.0000704.s004.docx]

# Supplementary File 4: Full description of included studies

| Study | Country | Context | Collection | Storage | Access |
| --- | --- | --- | --- | --- | --- |
| Aldekhyyel 2018 | United States | Single Centre | Television-based interactive patient care system | Univeristy of Minnesota Research Clinical Data repository and linked to in patient records through electronic health records | Electronic health records |
| Anthony 2021 | Canada | Multicentre | PROM portal | Not stated | Not stated |
| Barthel 2016 | Germany | Multicentre | Kids-CAT | Not stated | Kids-Cat report available to clinicians (printed) |
| Bele 2022 | Canada | Multicentre | KidsPRO | Within KidsPRO | KidsPRO application for children and families and within electronic health records for clinicians |
| Bower 2020 | United States | Single Centre | Pen and Paper | Smart forms in Epic | Electronic health records |
| Carberry 2016 | United States | Single Centre | Custom forms/Outpatient note templates (in electronic health records) | In EHR using outpatient note templates to ensure that outcomes are measures in a discrete mineable and reportable elements. | Electronic health records |
| Cheng 2022 | United States | Single Centre | Not stated | Not stated | Not stated |
| Chua 2023 | Singapore | Single Centre | Quick response codes | Not stated | Not stated |
| Cunningham 2018 | United States | Single Centre | Web-based data capture | Directly linked to electronic health records | Electronic health records |
| Eilander 2016 | Netherlands | Multicentre | Online survey | Not stated | Not stated |
| Engelen 2010 | Netherlands | Single Centre | QLIC-ON | Not stated | QLIC-ON profile access on the web |
| Engelen 2012 | Netherlands | Single Centre | QLIC-ON | Not stated | QLIC-ON profile access on the web |
| Fischer 2020 | Not stated | Multicentre | Kids-CAT | Not stated | Kids-Cat report available to clinicians (printed) |
| Fischmeister 2021 | Austria | Single Centre | Life App | Not stated | Not stated |
| Franklin 2021 | United States | Single Centre | PROMIS- part of electronic health records (electronic health records) | Not stated | Not stated |
| Gerhardt 2018 | United States | Single Centre | Electronic system | Electronic health records | Custom build PRO dashboard within electronic health records (results available at individual and population level) |
| Gmuca 2019 | United States | Single Centre | Online survey | Not stated | Not stated |
| Graham 2023 | Canada | Single Centre | KLIK | Stored in KLIK PROM portal | KLIK ePROfile retrieved from the website by clinicians |
| Gupta 2021 | Canada | Multicentre | Web-based platform | Scores are collected centrally in the Symptom management reporting database (SMRD) | Not stated |
| Gupta 2023 | Canada | Registry Network | Web-based platform | Scores are collected centrally in the Symptom management reporting database (SMRD) | Not stated |
| Hames 2016 | UK | Single Centre | Web-based platform (screening interface) | Stored in the system | Not stated |
| Hanmer 2021 | United States | Single Centre | Electronic survey linked to electronic health records, completed via tablets | Stored directly into electronic health records | Viewed in electronic health records |
| Haverman 2013 | Netherlands | Single Centre | KLIK | Stored in KLIK PROM portal | KLIK ePROfile retrieved from the website by clinicians |
| Haverman 2013 | Netherlands | Multicentre | KLIK | Stored in KLIK PROM portal | KLIK ePROfile retrieved from the website by clinicians |
| Haverman 2015 | Netherlands | Single Centre | KLIK | Stored in KLIK PROM portal | KLIK ePROfile retrieved from the website by clinicians |
| Haverman 2017 | Netherlands | Multicentre | KLIK | Stored in KLIK PROM portal | KLIK ePROfile retrieved from the website by clinicians |
| Haverman 2019 | Not stated | Not stated | KLIK | Stored in KLIK PROM portal | KLIK ePROfile retrieved from the website by clinicians |
| Hjollund 2023 | Denmark | Single Centre | Ambuflex- ePRO system but collection is done through eBoks which is a secure national email platform | Stored in the portal and can be anonymized and transferred for further analysis | PRO overview is presented inside the electronic health record system for clinicians (no patient access or portal). Patient is contacted if necessary |
| Knottnerus 2017 | Netherlands | Single Centre | KLIK | Stored in KLIK PROM portal | KLIK ePROfile retrieved from the website by clinicians |
| Kuhn 2022 | United States | Not stated | MyGeisinger patient portal | Electronic health records | Patient portal linked to electronic health records |
| Lassen 2023 | Denmark | Single Centre | Ambuflex- ePRO system but collection is done through eBoks which is a secure national email platform | Stored in the portal and can be anonymized and transferred for further analysis | PRO overview is presented inside the electronic health record system for clinicians (no patient access or portal). Patient is contacted if necessary |
| Leahy 2021 | Not stated | Not stated | Redcap | Downloaded from RedCap and processed with a excel macro for standardised reports to clinicians | Reports to clinicians from excel macros |
| Limperg 2012 | Netherlands | Single Centre | KLIK | Stored in KLIK PROM portal | KLIK ePROfile retrieved from the website by clinicians |
| Limperg 2013 | Netherlands | Single Centre | KLIK | Stored in KLIK PROM portal | KLIK ePROfile retrieved from the website by clinicians |
| Limperg 2017 | Netherlands | Single Centre | KLIK | Stored in KLIK PROM portal | KLIK ePROfile retrieved from the website by clinicians |
| Luijten 2020 | Netherlands | Not stated | KLIK | Stored in KLIK PROM portal | KLIK ePROfile retrieved from the website by clinicians |
| Marker 2019 | United States | Multicentre | REDCAP | Data stored into electronic health records and automatically pull scores from there | Viewed in electronic health records- automated scores and outcomes were documented in electronic health records |
| Mentrikoski 2018 | United States | Single Centre | Redcap | RedCap database | Not stated |
| Meryk 2021 | Austria | Single Centre | ePROtect | Downloaded from patient portal | ePROtect- web-based patient portal can view scores with descriptions and a health care professional interface to review the data from all patients |
| Meryk 2022 | Austria | Single Centre | ePROtect | Downloaded from patient portal | ePROtect- web-based patient portal can view scores with descriptions and a health care professional interface to review the data from all patients |
| Meryk 2022 | Austria | Single Centre | ePROtect | Downloaded from patient portal | ePROtect- web-based patient portal can view scores with descriptions and a health care professional interface to review the data from all patients |
| Meyerheim 2022 | Germany | Single Centre | MyPal-Child and MyPal-Carer app | Not stated | Reported data are visualised graphically for the medical staff via a web interface. |
| Morris 2023 | UK | Single Centre | myHealthE | Not stated | caregivers were presented with infographics based on their responses in the system within the |
| Munaretto 2021 | Italy | Single Centre | Google online form | Not stated | Not stated |
| Murphy 2011 | United States | Single Centre | Electronic outcomes rating form (e-ORF, a paper form that uses a digital pen for electronic entry) | A digital pen is used to transfer the data to the hospital service and a PDF version of the e-ORF is produced via FusionForm software. This PDF data is stored in another server. This PDF data can then converted into CSV. | Scores appear directly in the patient's medical record |
| Ng 2023 | United States and Canada | Registry Network | Electonic survey then application | Not stated | Not stated |
| Pasulo 2023 | Not stated | Single Centre | ePRO diary | In the software | Data extracted from the software by clinicians and patients |
| Perito 2021 | United States | Registry Network | Not stated | Data needed for registries required manual extraction and data transfer from electronic health records (EHR) into registry specific forms. | Not stated |
| Riedl 2022 | Austria | Single Centre | Life App | Not stated | Not stated |
| Riobueno-Naylor 2019 | United States | Multicentre | Tonic for Health Electronic Platform (online webpage and app) | Tonic cloud servers | Scores shown in either a laptop or paper printout to clinicians. |
| Robertson 2020 | UK | Single Centre | Qualtrics | Not stated | Not stated |
| Romo 2016 | United States | Single Centre | Tonic for Health Electronic Platform (online webpage and app) | Tonic cloud servers | Scores shown in either a laptop or paper printout to clinicians. |
| Ross 2021 | United States | Single Centre | Web-based portals | Electronic health records | Electronic health records |
| Schepers 2014 | Netherlands | Multicentre | QLIC-ON | Not stated | QLIC-ON profiles (web access) |
| Schepers 2016 | Not stated | Single Centre | KLIK | Stored in KLIK PROM portal | KLIK ePROfile retrieved from the website by clinicians |
| Schepers 2016 | Netherlands | Single Centre | KLIK | Stored in KLIK PROM portal | KLIK ePROfile retrieved from the website by clinicians |
| Schepers 2017 | Netherlands | Single Centre | KLIK | Stored in KLIK PROM portal | KLIK ePROfile retrieved from the website by clinicians |
| Schougaard 2019 | Denmark | Single Centre | Ambuflex- ePRO system but collection is done through eBoks which is a secure national email platform | Stored in the portal and can be anonymized and transferred for further analysis | PRO overview is presented inside the electronic health record system for clinicians (no patient access or portal). Patient is contacted if necessary |
| Sheikh 2021 | United States | Single Centre | Pen and Paper | Results were uploaded onto the electronic medical records on the day it was completed. | Electronic health records |
| Smyth 2021 | Not stated | Single Centre | Redcap | Not stated | Not stated |
| Spraggs-Hughes 2018 | United States | Single Centre | PROMIS-CAT | Electronic health records, clinicians have direct access | Electronic health records |
| Stratton 2022 | Canada | Single Centre | Redcap | Not stated | Not stated |
| Swales 2016 | UK | Single Centre | Paper collection then manually entrered onto the website | On the system for multi site analysis | Not stated |
| Taxter 2018 | United States | Single Centre | Web-based PROM portal | Built in clinic note templates to store in electronic health records and to capture the CARRA registry | Electronic health records, flowsheet function also means it can be shared with patients |
| Taxter 2022 | United States | Single Centre | Electronic collection but not specified | Electronic health records | Not stated |
| Teela 2019 | Netherlands | Multicentre | KLIK | Stored in KLIK PROM portal | KLIK ePROfile retrieved from the website by clinicians |
| Teela 2020 | Netherlands | Single Centre | KLIK | stored in KLIK PROM portal | KLIK ePROfile retrieved from the website by clinicians |
| Tollit 2019 | Australia | Single Centre | LimeSurvey | Parent, patient and in-clinic questionaiire responses (and scored summary data where relevant) are uploaded to patients' electronic medical record (EMR) and in the RCHGS clinical database | Accessed via electronic health records |
| Tyack 2020 | Australia | Single Centre | Qualtrics | Not stated | Not stated |
| Valles 2017 | Spain | Multicentre | Electronic collection but not specified | Not stated | Not stated |
| VanDerSluijsVeer 2013 | Netherlands | Not stated | KLIK | Not stated | Not stated |
| vanGorp 2021 | Netherlands | Single Centre | KLIK | Stored in KLIK PROM portal | KLIK ePROfile retrieved from the website by clinicians |
| VanMuilekom 2019 | Netherlands | Not stated | KLIK | Stored in KLIK PROM portal | KLIK ePROfile retrieved from the website by clinicians |
| vanMuilekom 2021 | Netherlands | Not stated | KLIK | Stored in KLIK PROM portal | KLIK ePROfile retrieved from the website by clinicians |
| vanMuilekom 2021 | Netherlands | Single Centre | KLIK | Stored in KLIK PROM portal | KLIK ePROfile retrieved from the website by clinicians |
| vanMuilekom 2022 | Netherlands | Not stated | KLIK | Stored in KLIK PROM portal | KLIK ePROfile retrieved from the website by clinicians |
| VanOers 2013 | Netherlands | Single Centre | KLIK | Stored in KLIK PROM portal | KLIK ePROfile retrieved from the website by clinicians |
| VanOers 2018 | Netherlands | Multicentre | KLIK | Stored in KLIK PROM portal | KLIK ePROfile retrieved from the website by clinicians |
| vanOers 2021 | Netherlands | Multicentre | KLIK | Stored in KLIK PROM portal | KLIK ePROfile retrieved from the website by clinicians |
| vanSonsbeek 2021 | Netherlands | Single Centre | Web-based questionnaire | Routine Outcome Monitoring (ROM) system or electronic health records | Results can be directly access from the ROM system or in the electronic health records (a day after) |
| Veltkamp 2022 | Netherlands | Single Centre | KLIK | Stored in KLIK PROM portal | KLIK ePROfile retrieved from the website by clinicians |
| Vuong 2022 | Netherlands | Single Centre | KLIK | Stored in KLIK PROM portal | KLIK ePROfile retrieved from the website by clinicians |
| Wang 2018 | United States | Not stated | Web based system | Not stated | Not stated |
| Wolfe 2014 | United States | Multicentre | PediQuest system | in the PediQUEST system | Printed feedback reports and e-mail alerts (PQ reports and emails) |
| Yao 2019 | United States | Single Centre | Not specified but online survey | Not stated | Not stated |
