# Supplementary material for: A scoping review of the electronic collection and capture of patient reported outcome measures for children and young people in the hospital setting
Source: PLOS Digit Health. 2025 Jan 6;4(1):e0000704. doi: 10.1371/journal.pdig.0000704 (PMC11703060; doi:10.1371/journal.pdig.0000704)
Supplement: Attachment — Submitted filename: ePROMs_revision_response.docx [file pdig.0000704.s005.docx]

*Thank you for your helpful review and the opportunity to revise this paper. Please see below our detailed responses and changes.*

Journal Requirements

1. Please provide separate figure files in .tif or .eps format.

*Figure 1 and 2 have now been converted into .TIFF files and uploaded as part of the submission.*

2. Please review your reference list to ensure that it is complete and correct. If you have cited papers that have been retracted, please include the rationale for doing so in the manuscript text, or remove these references and replace them with relevant current references. Any changes to the reference list should be mentioned in the rebuttal letter that accompanies your revised manuscript. If you need to cite a retracted article, indicate the article's retracted status in the References list and also include a citation and full reference for the retraction notice.

*This has now been checked to ensure that this is complete and correct.*

3. Could you please confirm that all data necessary to replicate your study’s findings is available in table 2, and we would also want to ask for the table represents the majority of the data, where the rest is if you can provide it.

*All the necessary data to replicate the study’s findings can be found in table 2 and the additional supplementary file 4 that we have now provided.*

Reviewer #1

*Thank you for your careful review and helpful comments.*

Reviewer #1: I congratulate Alarilla et al. on their submission, A scoping review of the electronic collection and capture of patient reported outcome measures for children and young people in the hospital setting.
This is a very comprehensive review of the current state patient report outcome measure assessments as recorded or stored in the electronic health record.

*Thank you for your positive feedback.*

The authors conducted a review twice, of 7 databases, first in February 2021 and again in April 2023, for studies that used electronic systems as part of patient reported outcome measure assessments in secondary and tertiary care settings. They focused on studies where data was collected from patients aged birth to 25 years, patient proxies, or clinicians working with the aforementioned patient population.
They identified 85 studies out of 372 that met their inclusion criteria.
The authors stated that the aim of their scoping review was to map the available evidence of the electronic collection and capture of PROMs used as part of standard care of children and young people (CYP) focusing on technical aspects.
• What are the electronic systems used for PROMs collection?
• What are the electronic systems used for the storage of PROMs data?
• How are PROMs data accessed electronically?
• How do the electronic collection, storage and access relate to one another?
The authors tables and accompanying text answer the above questions in detail, identifying uses, challenges and benefits of PROMs.
The authors found that though popular, there is significant variability in all aspects of use of PROM, from data collection, to storage, implementation and access.
They suggest, “further research to explore the technical considerations needed for implementation of electronic platforms, including creating interoperable solutions,” so the value of these assessments can be “maximized…and support the scalability of PROMs in routine care.”

Minor changes:
Abstract: The abstract appears to be incomplete. In the introduction, the last line appears to be cut off. “Yet little is known about…”
Last line, page 21 of manuscript, there is a period and comma.

*Thank you for bringing this to our attention, the last line of the abstract (“Yet little is known about the technical aspects of implementation including the electronic systems available for collection and capture and how this may influence the value of PROMs in routine care.”) has now been added to complete this.*

Comments/Questions:
1/ If you were to make suggestions about creating an ideal platform, what would you suggest? How would you address the challenges of universal digital literacy or access to such technology? When hybrid forms are used, there is such as burden on staff (transcribing paper responses, etc), and challenges to use the data, as identified in your manuscript. What would be an ideal platform in CYP to allow easy access, storage, use and implementation of this data?

*Exploring the ideal platform is beyond the scope of this review and should explore other aspects such as user interface, costs and integration to existing processes. However, the findings of this review suggests that when choosing a platform, the technical aspects of the platform and how the data are stored should also be considered. This includes exploring how platforms may use transport standards such as HL7 FHIR or how platforms collect PROMs data using common data standards to ensure that the PROMs data collected and captured are accessible, shareable and usable outside the individual platform. These interoperable solutions can help facilitate the ability to maximise the value of PROMs in routine care. The authors consider that this is an important comment to highlight and have now made changes to the discussion to reflect this (see page 22)*

*Furthermore, the findings suggests that the ideal platform should be able to offer flexibility in data collection (such as using pen and paper and electronic methods) and storing results electronically such as integration within electronic methods may be able to offer this flexibility. This can help address challenges of universal digital literacy or restricted access to technology to ensure that PROMs are beneficial across all patient groups. This has further been added to the discussion.*

One current conversation in digital health is how does one manage the overabundance of data? Do you envision this happening?

*The collection and use of PROMs as part of standard care of children and young people is a fast-evolving field. However currently the bottom-up fragmented approaches to implementation including the lack of consideration for interoperability may be hinder the usability and scalability of PROMs data collected in routine care. In addition, the self-report nature of PROMs data and the reliance on clinician engagement means that human factors affecting uptake and completion will continually be an influence that can impact the quality and quantity of data collected. Therefore, it is unlikely that there will be a large-scale PROMs data set for children and young people any time soon. However, the consideration of the technical aspects of the data collection such as the interoperability of the data even at early stages can help ensure that the data collected is easy to manage and is usable. We have further highlighted in our discussion that one of the benefits of considering interoperable solutions at early stages of the implementation is also to reduce the overabundance of unusable data (see page 22).*

Does AI and other machine learning platforms have a space in PROMs, while ensuring patient privacy?

*Although the use of AI and machine learning within PROMs is beyond the scope of this review, the authors recognise that the AI and machine learning may present innovative solutions for improving the use of health data including PROMs data but also presents new challenges such as privacy concerns. Further research is needed on the best policies for privacy and security when employing these tools which should also be considered when choosing a platform for PROMs collection and capture. This has now been added in our discussion (see page 22).*
